# Laparoscopic Versus Open Inguinal Hernia Repair: A Comparative Study

**DOI:** 10.7759/cureus.48619

**Published:** 2023-11-10

**Authors:** Renjul Pulikkal Reghunandanan, Azif Ali Usman, Shiraz Basheer, Lavith Kuttichi, Joicy Els Jojo, Muhamed Fawas Abdul Rasheed

**Affiliations:** 1 Department of General Surgery, Mount Zion Medical College, Adoor, IND; 2 Department of General Surgery, Muslim Educational Society (MES) Academy of Medical Sciences, Perinthalmanna, IND

**Keywords:** open mesh, laparoscopy, surgical repair, inguinal, hernia

## Abstract

Background: Inguinal hernia is a common surgical problem around the world. The two types of groin hernias are femoral hernias and direct and indirect inguinal hernias. The incidence rate is higher among males. This investigation intends to differentiate between open and laparoscopic methods of inguinal hernia surgery with respect to operative time, seroma formation, duration of hospitalization, and return to normal activity.

Materials and methods: This prospective observational study included 84 patients with unilateral, bilateral, direct, and indirect inguinal hernias, but excluded those who were unwilling to have surgery, were under 12, had comorbidities, or had complete and recurrent hernias. In the end, 42 underwent open, and 42 underwent laparoscopic repair. Visual analog scales were used for pain assessments. Chi-square and unpaired student T-tests were employed (p<0.05).

Results: Among the 84 individuals analyzed, 79 (94.04%) were male patients, with the majority of them falling between the ages of 41 and 55. In contrast to the open group of patients, the laparoscopic group experienced a significant increase in operative time with a highly significant statistical difference (p<0.0001), and the laparoscopic group experienced a significant decrease in post-operative pain score with an insignificant p-value. A significant statistical difference (p<0.005) was estimated among the laparoscopic and open groups of patients in terms of post-operative hospitalization. Returning to normal activities was significantly different for laparoscopic patients relative to the open group (p-value<0.001). With a high level of significance of p<0.001, laparoscopic hernia repair required less time to recover before returning to normal activities than open hernia repair (p<0.005).

Conclusion: In terms of decreased post-operative discomfort, shorter hospitalization, and an earlier return to activities, laparoscopic hernia repair has been found to be superior to open hernia repair, which is also known as Lichtenstein surgery. However, there was no discernible difference among the two groups with regard to post-operative problems, including seroma development and wound infections. To assess chronic discomfort and recurrence rates after laparoscopic hernia surgery, additional studies and extended follow-up are required.

## Introduction

Inguinal hernias are common and have become common surgical problems; over the past few decades, their mode of repair has changed, and new studies are constantly being conducted in this area [1]. The two types of groin hernias are femoral hernias and direct and indirect inguinal hernias [2]. A patent internal inguinal ring allows the peritoneum (with or without peritoneal contents) to protrude laterally to the inferior epigastric veins, resulting in the most common type of inguinal hernia, known as an indirect hernia. Hernioplasty is a frequent general surgery procedure that has historically been managed using open techniques; however, in the past two decades, the advent of minimally invasive surgery has transformed the scenario [3,4]. Men may develop hernias along the spermatic cord that can eventually reach the scrotum, whereas hernias in females may follow the course of the round ligament into the labia majora [5]. With a prevalence of 1.7% across the board and 4% in people over 45, abdominal wall hernias are frequently seen. In total, 75% of abdominal wall hernias are inguinal hernias, which occur in 27% of males and 3% of females during the course of a lifetime [6].

The Lichtenstein tension-free mesh repair, in which the mesh is positioned anteriorly between the external and internal oblique aponeuroses, is regarded as the standard mesh repair technique [7]. The plug-and-patch method, the Gilbert Prolene Hernia System (PHS) bilayer-linked device repair, and the placement of an open preperitoneal mesh through an inguinal incision following hernia reduction are other open mesh techniques, but they are not recommended in general by the current guidelines [8,9]. Two of the most popular laparoscopic (keyhole) procedures are transabdominal preperitoneal repair (TAPP) and totally extraperitoneal repair (TEP). Recently, laparoscopic procedures have become more common, with some surgeons appreciating the decreased frequency of chronic post-operative pain. However, concerns remain regarding the potential risk of recurrence following TEP repair [10].

The intention of this study is to evaluate the open and laparoscopic approaches to inguinal hernia surgery in terms of surgical duration, seroma formation, length of hospitalization, and recovery time before returning to regular activity.

## Materials and methods

A prospective comparison study was performed on individuals hospitalized in the Department of General Surgery at the Muslim Education Society (MES) Medical College in Perinthalmanna, India, who had inguinal hernias that were clinically determined for surgical repair. The information was gathered over the course of a year, from January 1 to December 31, 2019. The study was approved by the MES Medical College (approval number: IEC/MES/16/2018). Patients with uncomplicated bilateral and unilateral inguinal hernias who agreed to laparoscopic (TEP) and open hernia surgery were included in the study. Patients who refused surgery, were younger than 12 years old, had concomitant conditions (type 2 diabetes mellitus, hypertension, coronary artery disease, or tuberculosis), or had complete and recurrent hernias were excluded. Following a thorough analysis and investigation, data from every inguinal hernia diagnosis that met the inclusion and exclusion criteria were considered. Written consent to participate in the study was obtained.

Sample size

The sample size was determined with the following formula from the study by Sudarshan et al. [11].

n = Z21-α/2 [ 2SP2] / d2

Sp = S1 + S2 / 2

where S12: standard deviation in the open group =5.4; S22: standard deviation in the lap group = 4.1; SP2: pooled standard deviation; d: precision = 2; 1-α: significance level = 95%. Hence, the estimated sample size was 42 in each group.

Procedure

Two groups of patients undergoing open and laparoscopic repairs were observed for the study. The study comprised patients with uncomplicated bilateral and unilateral inguinal hernias who agreed to laparoscopic (TEP) and open hernia surgery. The individuals were brought in for surgery, and the duration of the procedure was recorded. Patients were treated with appropriate analgesics following surgery, mostly non-steroidal anti-inflammatory drugs (NSAIDS) with no contraindication, and the pain was evaluated utilizing the Visual Analogue Scale (VAS). An analysis of pain was done two days, one week, and four weeks after surgery. After four hours of recovery from surgery, oral feedings were initiated for all patients. The sutures were removed at the patient's first follow-up appointment a week later. Following that, they were observed for a further six months to assess seroma formation, wound infection, and their return to normal activities.

Open Hernia Repair

Open hernia repair was carried out; prior to surgery, all patients undergoing open hernia repair received prophylactic antibiotics, as recommended by clinical guidelines, to reduce the risk of surgical site infection. General anesthesia was administered to patients undergoing open hernia repair. Anesthesia was administered by an anesthesia team consisting of an anesthesiologist and certified nurse anesthetists. The surgical procedures were performed by a team of experienced surgeons, including senior surgeons and surgical residents under supervision. The specific designation of the surgeon (senior or resident) was noted in the patient records. The open hernia repair procedure followed the principles outlined by Parviz K. Amid in the Lichtenstein technique. The key steps of the open hernia repair included a standard inguinal incision. The inguinal canal and hernia sac were carefully dissected and isolated. A suitable mesh, typically made of polypropylene, was placed over the defect. The mesh was secured in place using non-absorbable sutures or tacks, following standard fixation techniques. The incision was closed using an appropriate skin closure technique, such as subcuticular sutures or skin staples.

Laparoscopic Hernia Repair

Laparoscopic hernia repair (TEP) was carried out similarly to open hernia repair. Patients undergoing laparoscopic hernia repair received prophylactic antibiotics before surgery to prevent infection. Laparoscopic hernia repair was performed under general anesthesia, and administered by the anesthesia team. The laparoscopic procedure was performed by experienced surgeons, including senior surgeons and surgical residents under supervision. The specific surgeon's role was documented in patient records. The laparoscopic hernia repair (TEP) procedure involved the following steps. Balloon dissection was employed to create the preperitoneal space. Trocar ports were positioned according to standard technique, allowing access to the inguinal region. A suitable mesh, often composed of polypropylene or other materials, was placed in the preperitoneal space. The mesh was secured using appropriate techniques, such as self-fixating mesh or tack fixation. The trocar incisions were closed in accordance with standard laparoscopic closure methods.

After a recovery period of approximately four hours following surgery, all patients were allowed to resume oral feedings as tolerated. Patients were encouraged to ambulate and engage in light physical activity as soon as possible after surgery to prevent complications and aid in the recovery process.

Statistical analysis

Data were entered into an Excel spreadsheet (Microsoft Corporation, Redmond, Washington, United States), and descriptive and inferential statistical analyses were performed using SPSS Statistics for Windows, Version 16.0 (Released 2007; SPSS Inc., Chicago, United States). Both quantitative data (mean and standard deviation) and qualitative data (proportions and percentages) were presented accordingly. The Chi-square test was used to analyze the proportional difference. The unpaired student T-test for parametric data was used to analyze how the means of the groups differed from one another. Tests were given a 95% (p<0.05) level of significance.

## Results

The 84 patients analyzed included 79 (94.04%) male patients, most of whom were 41-55 years old. In this sample, the average age was 47.8 ±14.3 years (Table 1).

**Table 1:** The demographic characteristics of the recruited patients n: Number of patients; %: Percentage

Age in groups (years)	Female n (%)	Male n (%)
11-25	1 (20)	4 (5.1)
26-40	3 (60)	18 (22.8)
41-55	1 (20)	32 (40.5)
56-70	0	20 (25.3)
71-85	0	5 (6.3)

The duration of open surgery for bilateral direct inguinal hernia repairs was recorded as 58.75 ± 6.8 minutes, whereas laparoscopic surgery took 107.42 ± 8.9 minutes. In contrast, the duration of indirect surgery for the same was found to be 61.21 ± 3.87 minutes. As a result, compared to bilateral open mesh surgery, laparoscopic hernia repair in situations with bilateral hernias took substantially longer to complete.

In addition, the mean durations for repairs in the case of unilateral direct hernias were found to be 47.14 ± 7.2 minutes and 84.24 ± 13.8 minutes for open hernia repair and laparoscopic hernia repair, respectively. The average time of laparoscopic repairs for unilateral indirect hernias was recorded as 89.94 ± 9.53 minutes, while open hernia repairs required approximately 52.51 ± 5.61 minutes. As a result, whether the hernia was indirect or direct, unilateral hernias required a significantly greater duration of time to repair laparoscopically, with a p-value<0.001 showing high statistical significance (Table 2).

**Table 2:** Inguinal hernia classifications with associated operative times for open and laparoscopic procedures **Statistically highly significant n: Number of patients; %: Percentage

Type of hernia	n (%)	Operation time (minutes)		p-value
		Laparoscopic surgery (mean ± SD)	Open surgery (mean ± SD)	
Bilateral direct	10 (11.9%)	107.42 ± 8.9	58.75 ± 6.8	< 0.001**
Bilateral indirect	2 (2.3%)	112.5 ± 5.73	61.21 ± 3.87	
Right direct	20 (23.8%)	84.24 ±13.8	47.14 ± 7.21	< 0.001**
Left direct	8 (9.5%)			
Right indirect	28 (33.3%)	89.94 ± 9.54	52.51 ± 5.61	
Left indirect	18 (21.4%)			

The number of seromas after one week of laparoscopic hernia repair was three, compared to nine after one week of open hernia surgery and nil after four weeks, which was not statistically significant. Laparoscopic surgery patients (7.1%) reported less post-operative discomfort at weeks one and four than those who underwent open surgery, which was shown to be statistically insignificant (Table 3). There were no wound infections reported for both surgeries.

**Table 3:** Post-operative complications reported during follow-up p-value>0.005, statistically insignificant n: Number of patients; %: Percentage; NA: Not applicable

Post-operative Complications	At week 1, n (%)		At week 4, n (%)		p-value
	Laparoscopy	Open	Laparoscopy	Open	
Pain	3 (7.1% )	14 (33.3% )	0	5 (11.9% )	0.687
Seroma formation	3 (7.1 %)	9 (21.4 %)	0	0	0.541
Wound infection	0	0	0	0	NA

The recovery times for open and laparoscopic repairs were 14.5 days and 7 days, respectively (Table 4). Laparoscopic hernia repair required less recovery time before getting back to regular activities than open hernia repair, with high levels of significance (p<0.001 and p<0.005, respectively).

**Table 4:** Days of hospitalization after surgery and return to normal life activities *statistically significant, ** statistically highly significant

Type of surgery undergone	Days (Mean ± SD)	
	Hospitalization	Returned to normal life activities
Laparoscopy	1.9 ± 0.29	7 ± 1.9
Open Repair	2.21 ± 0.41	14.5 ± 1.7
p-value	<0.005*	< 0.001**

## Discussion

The majority of study participants were male (94%), and 40% of them were between the ages of 41 and 55. We documented 84 inguinal hernia repairs, including both open (n=42) and laparoscopic (n=42) hernia repairs. While Charles et al. [12] stated that 93.2% of all their cases were male, Gupta et al. [13] reported that inguinal hernia occurs 96% more frequently in men, demonstrating a low prevalence in females. The average age of study participants was 47.8 ±14.3 years. A total of 28 (33%) of the 84 instances had right indirect hernias, whereas bilateral (2%) were rare. In the current study, it was shown that the average operating times for open and laparoscopic hernia repairs were 47.14± 7.2 minutes and 84.24 ±13.8 minutes, respectively, for unilateral direct hernias, whereas 52.51 ± 5.61 minutes and 89.94 ± 9.54 for unilateral indirect hernias. Therefore, compared to open surgery, which was also consistent with other studies [14], the time needed to execute a laparoscopic hernia repair in cases of unilateral hernia, whether indirect or direct, was considerably longer (p<0.001). The average time to repair a bilateral direct inguinal hernia using open surgery was 58.75±6.8 minutes, while adopting a laparoscopic approach took 107.42± 8.9 minutes; in bilateral indirect hernias, it took 61.21± 3.87 minutes and 112.5± 5.73 minutes, respectively. Due to this, bilateral hernia laparoscopic repairs took longer than bilateral open mesh surgery. These findings are consistent with previous studies [15-17], but they contrast with other studies that showed no statistically significant difference in the mean operative times between the two groups [18,19]. The open repair (Lichtenstein technique) in our study caused more post-operative pain than the laparoscopic repair (TEP), which (p-value<0.5) may be related to the considerable dissection required for tissue repair. As a result, since it is not statistically significant, the number of days of post-operative pain experienced after Lichtenstein's repair and a laparoscopic repair are not comparable. This study was in line with that of Shah et al. [20]. Patient early mobilization and improved post-operative satisfaction are both enhanced by minimal post-operative discomfort [21].

In accordance with the current study, the average hospital stays following open and laparoscopic hernia repairs are 2.21 ± 0.41 days and 1.9 ± 0.29 days, respectively. The study observed that patients who underwent laparoscopic hernioplasty had significantly shorter hospital stays compared to those who underwent open surgery (p<0.001). Specifically, the mean hospital stay for the laparoscopic group was 1.56 days, while for the open group, it was 1.9 days (p=0.002) [22]. In open hernia surgery, there were nine cases of seroma development, whereas laparoscopic hernia repair resulted in three cases (p>0.05). This difference in seroma occurrence could potentially be associated with the use of a larger incision and/or the presence of a larger hernial sac. In the current study, laparoscopic and open hernia repairs took 14.5 days and seven days, respectively, to allow patients to return to their regular jobs. When compared to other studies [23], laparoscopic hernia repair took considerably less time to recover than open repair (p <0.001). The results of other investigations were ambiguous in comparison with this [10,24].

The study has some limitations such as a relatively small sample size of 84 patients, which may limit the generalizability of the findings to a broader population. This investigation adopted a prospective observational design, which inherently lacks the experimental control of randomized controlled trials. As a result, the findings may be influenced by confounding variables that were not considered in the study. The study primarily focused on short-term outcomes, including post-operative pain and return to normal activities. Long-term outcomes, such as chronic discomfort and recurrence rates, were not extensively evaluated, necessitating additional research with extended follow-up periods to draw more comprehensive conclusions. The study may be limited in its external validity as it was conducted in a single center. Different healthcare settings and patient populations may yield different results.

## Conclusions

The investigation aimed to draw parallels between the effectiveness of open surgery with laparoscopic repair and any potential negative effects. Because of its lower risk of complications, shorter recovery time, and shorter hospital stay, laparoscopic hernia repair is preferred over Lichtenstein surgery. In regard to post-operative problems such as seroma formation and wound infections, all groups exhibited similar outcomes without significant differences. Laparoscopic repair is thought to be a preferable option for inguinal hernia correction, despite its own drawbacks, including a longer operative time. To assess chronic discomfort and recurrence rates after laparoscopic hernia surgery, additional studies and extended follow-up are required.
